# Differentially Private Hierarchical Spectral Clustering

**DOI:** 10.3390/e28090963

**Published:** 2026-08-27

**Authors:** Mohamed Seif Mohamed, Andrea J. Goldsmith

**Affiliations:** 1 Department of Electrical and Computer Engineering, Princeton University, Princeton, NJ 08544, USA; 2 Department of Computer Science and Engineering, Oakland University, Rochester, MI 48309, USA; 3 Department of Electrical and Computer Engineering, Stony Brook University, Stony Brook, NY 11794, USA

**Keywords:** hierarchical spectral clustering, differential privacy, random graph models, stochastic block models, graph Laplacian, noisy power iteration, eigengap

## Abstract

We study hierarchical spectral graph clustering under edge differential privacy (DP) through the lens of iterative eigenvector estimation on adjacency matrices. We propose a differentially private recursive spectral framework, where each binary partition is obtained via a rank-one noisy power method applied to induced adjacency sub-matrices. At each iteration, carefully calibrated Gaussian noise is injected into the matrix–vector multiplication, ensuring (ε,δ)-edge DP under cumulative privacy accounting across both power iterations and recursive hierarchy levels while preserving the essential convergence properties of the classical power method. We provide a non-asymptotic analysis of the resulting noisy iterations, characterizing the trade-off between privacy and accuracy via explicit bounds on the eigenvector estimation error. In particular, we quantify how the noise variance, number of iterations, eigengap, and hierarchy depth jointly influence the accuracy of each recursive split and the overall clustering performance. Empirical evaluations on synthetic and real-world networks validate the theoretical predictions and demonstrate that the proposed method achieves strong multi-scale clustering performance under meaningful privacy budgets.

## Dedication

*It is an honor and pleasure to dedicate this article to H. Vincent Poor on the occasion of his 75th birthday. Vince has been an incredible inspiration to me and to so many others throughout his long and distinguished career. Vince is an exceptional scholar who has pioneered new and profoundly impactful results spanning signal processing, wireless communications and networks, power systems, as well as information-theoretic security and privacy. Vince is an outstanding collaborator through his unparalleled brilliance, unique wisdom, keen insight, and deep knowledge. He is an invaluable mentor and supporter to students and colleagues alike, an award-winning teacher, and a tireless contributor to the broad technical community. Vince’s transformational leadership at Princeton, in the IEEE, and in the NAE has profoundly improved all of these organizations. I have greatly enjoyed and learned much from my many collaborations with Vince, including our three books, dozens of research papers, and more than a dozen jointly supervised students and postdocs. I have also benefitted tremendously from his mentorship and support. Most importantly, Vince is a dear and treasured friend who has greatly enriched my life and that of my family. Happy Birthday, Vince. I look forward to many more years of collaboration, celebrations, and friendship. With love, Andrea Goldsmith.*

## 1. Introduction

Many real-world networks exhibit hierarchical community structure, in which groups of nodes are recursively subdivided into smaller, more refined subgroups across multiple scales. Such multi-resolution organization is ubiquitous in practice, arising naturally in social networks [1] (e.g., communities within communities), biological systems [2] (e.g., gene regulatory or protein interaction networks), and information networks [3] (e.g., topic hierarchies in citation or document graphs). This structure extends beyond flat partitions by capturing the inherent nested relationships present in complex systems. While clustering in random graph models has been extensively studied [4], the principled characterization and statistical recovery of hierarchical structure remain not as well understood. The seminar work of [5] provides a formal framework for defining hierarchical communities and highlights the fundamental challenges in reliably detecting multi-level organization in networks.

Hierarchical clustering provides a natural framework for modeling such multi-scale structure. As a fundamental class of unsupervised learning methods [6], it constructs a nested sequence of partitions, progressively refining clusters into smaller subclusters. In contrast to flat clustering approaches such as *k*-means [7], which produce a single partition of the data, hierarchical methods yield a tree-structured representation (dendrogram) that enables analysis at multiple levels of granularity. This paradigm has found widespread use in applications such as phylogenetic tree reconstruction in computational biology, document and topic hierarchies in information retrieval, image segmentation and computer vision [8], and market segmentation and recommendation systems. More recently, hierarchical clustering has emerged as a key tool in large language model (LLM) pipelines for long-context understanding and summarization. In particular, systems such as CLIO [9,10] leverage hierarchical clustering to organize text into multi-level semantic groups, enabling recursive summarization where coarse representations are refined into finer-grained descriptions. This hierarchical organization improves scalability and coherence when processing long documents, aligning naturally with the multi-resolution structure inherent in textual data.

Hierarchical clustering algorithms can be broadly classified into two categories: (1) *agglomerative* and (2) *divisive* methods. Agglomerative hierarchical clustering (e.g., Chameleon [11] or CURE [12]) proceeds in a *bottom-up* manner, starting with each node as an individual cluster and iteratively merging the most similar pairs of clusters according to a predefined linkage criterion (e.g., single, complete, or average linkage). While these methods are conceptually simple and widely used in practice, they are often *sensitive to noise and local perturbations*, and their greedy merging steps make it challenging to establish rigorous theoretical guarantees, particularly in stochastic graph models. In contrast, *divisive* methods adopt a *top-down* approach, beginning with the entire graph and recursively partitioning it into smaller subgraphs. Each split aims to separate the nodes into more homogeneous groups, naturally producing a hierarchical structure aligned with multi-scale community organization. In this paper, we focus on *divisive approaches*, and in particular on *recursive spectral methods*, where each partition is obtained via a spectral decomposition (e.g., leading eigenvector of a graph adjacency matrix). This choice is motivated by both *statistical and computational considerations*; spectral methods admit *strong theoretical guarantees* under random graph models and enable efficient implementations.

### 1.1. Privacy

Network data, such as social, communication, and information graphs, often encode sensitive relational patterns at multiple levels of granularity. In hierarchical settings, this sensitivity is further amplified, as interactions within and across communities may reveal fine-grained structural information about individuals and groups. Ensuring privacy in such multi-scale graph analysis is therefore of central importance.

Differential privacy (DP) [13] provides a principled framework for privacy-preserving data analysis, offering formal guarantees that the inclusion or removal of a single element induces only a limited change in the output distribution of a mechanism. In graph settings, two primary notions have been studied [14,15]: *edge DP*, which protects individual relationships by requiring stability under the addition or removal of a single edge, and *node DP*, which protects an entire node along with all of its incident edges. For clustering and community detection, edge DP is sufficient to obtain privacy across all network interactions since the interactions are only on the edges, as these tasks fundamentally rely on pairwise interactions [16,17,18,19,20]. However, most existing works focus on *flat* clustering, where a single partition of the network is recovered. In contrast, hierarchical settings require recursive access to induced subgraphs, which can compound privacy leakage across multiple levels. As a result, preserving privacy in hierarchical clustering necessitates careful control of both noise injection and the allocation of the privacy budget across iterations and depths of the hierarchy. While DP has been extensively studied for graph tasks such as subgraph counting [21], PageRank [22,23], and synthetic graph generation [24,25,26,27,28,29,30], the problem of *hierarchical clustering under privacy constraints* remains largely unexplored. This gap is particularly significant in modern applications, where multi-scale or tree-structured organization more accurately reflects the underlying structure than flat partitions.

### 1.2. Related Work

The closest work to our study is [31], which proposes an ε-edge DP clustering algorithm with polynomial-time complexity. Their approach constructs a privacy-preserving partition by perturbing graph statistics while maintaining sufficient separation between clusters, and it establishes theoretical guarantees for accurate community recovery under edge-level privacy constraints. However, their method does not explicitly exploit the *spectral* structure of the graph, which is known to offer strong statistical and computational advantages.

Imola et al. primarily formulated private hierarchical clustering through Dasgupta’s cost objective and established approximation guarantees and lower bounds for this objective. In the HSBM setting, their approach first privately recovers the underlying communities and subsequently constructs a hierarchy from noisy inter-community similarities. Although spectral techniques are employed within their community-recovery procedure, spectral estimation is not itself the recursive hierarchical splitting mechanism.

In contrast, our work focuses directly on the privacy–utility behavior of recursive spectral partitioning. At each node of the hierarchy, we apply a Gaussian-perturbed power iteration to the corresponding induced adjacency matrix and analyze how the resulting spectral estimation error affects the recursive split. This enables us to explicitly characterize the roles of the local eigengap, cluster size, number of power iterations, privacy-budget allocation, and hierarchy depth. Our guarantees therefore concern eigenvector accuracy, split accuracy, and conditions for exact recovery of the target hierarchy, rather than approximation of Dasgupta’s objective. Hence, our results are complementary to those of Imola et al. [31]. Their work addresses private hierarchical clustering from an objective approximation perspective, whereas ours develops a privacy and recovery analysis tailored to recursive spectral hierarchical clustering. Table 1 summarizes the key differences.

Spectral clustering [32,33] is a widely used and computationally efficient approach for graph partitioning, with well-established guarantees under a range of random graph models. Classical results show that leading eigenvectors of adjacency or Laplacian matrices recover latent communities under suitable separation conditions [4,34,35], with extensions to sparse and degree-heterogeneous settings [36,37,38]. Despite these advances, the interplay between spectral methods and privacy constraints remains relatively underexplored, particularly in hierarchical settings. Existing works on private spectral clustering focus primarily on *flat* community recovery. For example, Hehir et al. [28] analyzed randomized response mechanisms and showed that accurate recovery requires stronger connectivity conditions than in the non-private setting. Suppakitpaisarn et al. [20] studied locally private algorithms but restricted attention to two-community models and specific perturbation mechanisms.

Recent work, including our prior study [18], explored alternative privacy mechanisms for spectral clustering, such as randomized response and output perturbation tailored to graph settings, and analyzed their impact on clustering performance. However, these approaches remain limited to *flat* community detection and do not address multi-scale structure. In contrast, we develop a framework based on *recursive spectral methods* for hierarchical clustering under differential privacy. By employing noisy power iterations at each level, our approach captures multi-scale community structure while enabling controlled allocation of the privacy budget across recursive splits. This setting introduces new challenges, as estimation errors can propagate across levels of the hierarchy and privacy guarantees must be accumulated across both iterations and recursive partitions. To the best of our knowledge, there is currently no unified framework that characterizes these effects under edge DP. Our work bridges this gap by providing a principled analysis of *hierarchical spectral clustering under edge DP*, explicitly quantifying how privacy noise interacts with spectral perturbation and recursive splitting across multiple levels of the hierarchy. Our main contributions in this paper can be summarized as follows:

- Private hierarchical spectral framework: We introduce a hierarchical spectral clustering algorithm under (ε,δ)-edge DP where each recursive partition is obtained via a noisy spectral method.
- Noisy power iteration under DP: We adapt the classical power method by injecting Gaussian noise at each iteration and show that the resulting mechanism satisfies (ε,δ)-edge DP under composition across both iterations and hierarchy levels.
- Eigenspace perturbation analysis: We derive non-asymptotic bounds on the deviation of the estimated eigenspaces, explicitly quantifying the effects of noise variance, graph sparsity, and the iteration depth.
- Privacy–accuracy trade-offs: We identify the fundamental scaling laws governing the interplay between privacy parameters, graph structure, and sample complexity in hierarchical clustering.

Notation. We use bold uppercase letters to denote matrices (e.g., A) and bold lowercase letters for vectors (e.g., a). Let $A\in R^{n\times n}$ denote a symmetric matrix capturing pairwise relationships between nodes (e.g., an adjacency or similarity matrix). For any subset S⊆V, we denote by $A_{S}\in R^{\left|S|\times |S\right|}$ the local matrix obtained by restricting A to the rows and columns indexed by *S*. The notation Bern(p) denotes a Bernoulli random variable with a success probability *p*. For asymptotic analysis, we write f(n)=o(g(n)) if $\lim_{n\to \infty }f\left(n\right)/g\left(n\right)=0$. Similarly, f(n)=O(g(n)) indicates the existence of a constant C>0 such that |f(n)/g(n)|≤C for all *n*, and f(n)=Ω(g(n)) indicates the existence of a constant c>0 such that |f(n)/g(n)|≥c for all *n*. The notation used throughout the paper is provided in Table 2.

Paper Organization. The remainder of the paper is organized as follows. Section 2 introduces the problem formulation, provides the necessary preliminaries, and presents our main theoretical results. Section 3 presents numerical experiments that validate our theoretical findings. Additional technical details and proofs are provided in the Appendix A.

## 2. Problem Formulation and Preliminaries

### 2.1. Graph Model and Hierarchical Structure

Let G=(V,E) be an undirected graph with a vertex set V={1,…,n} and adjacency matrix $A\in {\left\{0,1\right\}}^{n\times n}$ satisfying $A=A^{⊤}$ and $A_{ii}=0$ for all i∈[n]. We assume that the vertex set admits an underlying hierarchical clustering structure represented by a binary tree $T^{★}$ of a depth $L=\log_{2}k$, whose leaves correspond to the *k* ground-truth communities. For each internal node S⊆V in $T^{★}$, let $\left(S^{+},S^{-}\right)$ denote its two children, where

$$S=S^{+}\cup S^{-},\;S^{+}\cap S^{-}=\emptyset .$$

Thus, the hierarchy is recovered once all local binary splits are recovered correctly.

#### 2.1.1. Global Block Structure

Let $V=C_{1}\cup \cdots \cup C_{k}$ denote the leaf partition. After permuting the vertices so that nodes belonging to the same leaf community appear contiguously, the adjacency matrix A admits the block representation as follows:

(1) $$A=\left[\begin{array}{cccc} A_{11} & A_{12} & \ldots & A_{1k}\\ A_{21} & A_{22} & \ldots & A_{2k}\\ \vdots & \vdots & \ddots & \vdots \\ A_{k1} & A_{k2} & \ldots & A_{kk} \end{array}\right],$$

where $A_{rs}\in {\left\{0,1\right\}}^{|C_{r}\left|\times \right|C_{s}|}$ records the edges between communities $C_{r}$ and $C_{s}$.

#### 2.1.2. Hierarchical Stochastic Block Model (HSBM)

**Figure 1:**
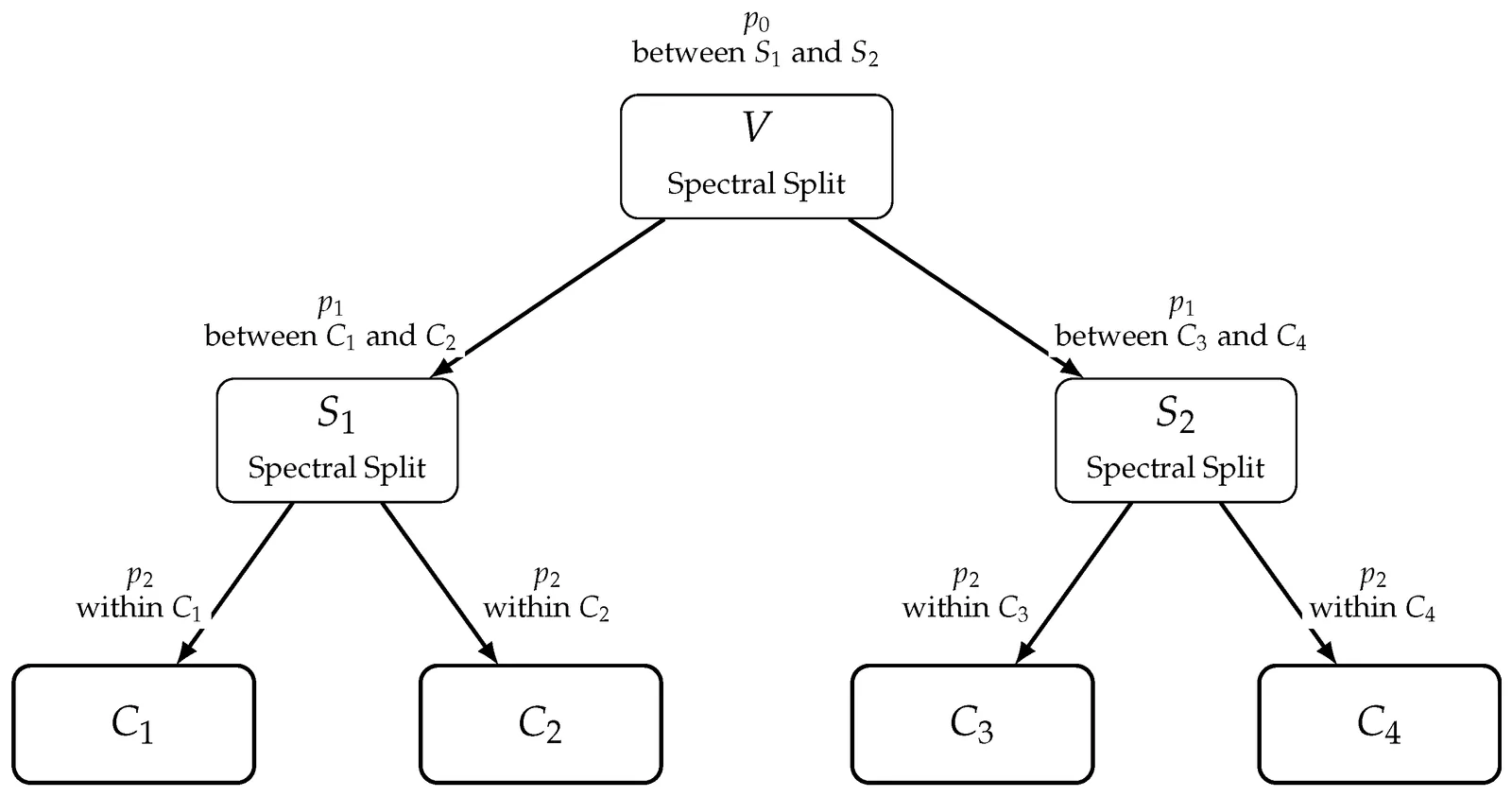
Hierarchical spectral clustering via recursive bipartitioning on a hierarchical graph. At each internal node, a private rank-one noisy power iteration is used to perform a binary spectral split. The edge probabilities depend on the level of the hierarchy: $p_{0}$ across the two top-level groups, $p_{1}$ across sibling leaf communities within the same parent group, and $p_{2}$ within each leaf community, typically with $p_{2}>p_{1}>p_{0}$.

For analytical tractability, we model the graph as an instance of a hierarchical stochastic block model. Vertices are associated with labels $\left\{z_{i}\right\}$ induced by a rooted tree $T^{*}$ of a depth *L*. For any pair (i,j), let d(i,j) denote the depth of their lowest common ancestor. Edges are generated independently with a probability $p_{d(i,j)}$, where $\left(p_{0},\ldots ,p_{L}\right)$ is a non-decreasing sequence. This model captures a multiscale clustering structure in which connectivity strength reflects hierarchical proximity. Figure 1 provides an illustration of the hierarchical structure and the associated connection probabilities.

#### 2.1.3. Local Spectral Operator

For every node S⊆V, let $A_{S}\in R^{m\times m}$ denote the adjacency submatrix induced by *S*, where m:=|S|.

For every node S⊆V, let $A_{S}\in R^{m\times m}$ denote the adjacency submatrix induced by *S*, where m:=|S|. We work directly with the raw local adjacency matrix $A_{S}$ and do not apply centering or any further modification. This choice preserves the clean sensitivity structure required by the noisy power method.

Let $u_{S}^{★}\in R^{m}$ denote the dominant unit eigenvector of $A_{S}$ targeted by the power iteration. For the exact recovery analysis, we make the explicit structural assumption that $u_{S}^{★}$ is sign-consistent with the target binary partition at node *S*. In the balanced idealized setting considered here, we assume

(2) $$\begin{array}{c} u_{S,i}^{★}\in \left\{+\frac{1}{\sqrt{m}},-\frac{1}{\sqrt{m}}\right\},\;i\in S, \end{array}$$

up to an overall sign flip. We emphasize that this is an assumption on the graph instances covered by our exact recovery result and is not asserted to hold for the leading eigenvector of an arbitrary adjacency matrix.

We further define the local eigengap and contraction ratio by

(3) $$\Delta_{S}:=\lambda_{1}\left(A_{S}\right)-\lambda_{2}\left(A_{S}\right),$$

and

(4) $$\rho_{S}:=\frac{\lambda_{2}\left(A_{S}\right)}{\lambda_{1}\left(A_{S}\right)}<1.$$

#### 2.1.4. Local Misclassification Rate

Given a candidate unit vector $x_{S}\in R^{m}$ obtained from a recovery algorithm, we quantify the local split error as follows:

(5) $${\mathrm{err}}_{S}:=\frac{1}{m}\cdot \min_{s\in \{\pm 1\}}\left|\left\{i\in S:\mathrm{sign}\left(x_{S,i}\right)\neq \mathrm{sign}\left(s\;u_{S,i}^{★}\right)\right\}\right|.$$

Alternatively, the overlap rate is obtained as follows:

(6) $$\begin{array}{c} \mathrm{overlap}\;{\mathrm{rate}}_{S}=1-{\mathrm{err}}_{S}. \end{array}$$

In addition to accurate split recovery, we require that the recovery algorithm protects the individual relationships (i.e., the edges in the graph), which we discuss in detail next.

### 2.2. Differential Privacy on Graphs

We focus on the notion of *edge* privacy [27], where two graphs *G* and $G^{\prime }$ are said to be neighbors, denoted by $G∼G^{\prime }$, if they share the same vertex set but differ in exactly one edge. The formal definition is given next.

**Definition 1** 
((ε,δ)-edge DP [14])**.** *A (randomized) clustering algorithm A as a function of a graph G satisfies (ε,δ)-edge DP for some $\varepsilon \in R^{+}$ and δ∈[0,1] if, for all pairs of adjacency matrices G and $G^{\prime }$ that differ in one edge and any measurable subset S⊆Range(A), we have*

$$\begin{array}{c} \mathrm{Pr}(A(G)\in S)\leq e^{\varepsilon }\mathrm{Pr}(A\left(G^{\prime }\right)\in S)+\delta , \end{array}$$

*where the probabilities are computed only over the randomness in the clustering process. The setting where δ=0 is referred to as ε-edge DP. Here, larger values of ε or δ mean weaker privacy constraints and vice versa. Our ultimate goal is to design a private clustering algorithm that maintains high accuracy guarantees for the underlying communities while protecting individual relationships (i.e., the edges of the graph) in the graph.*

### 2.3. Recursive Rank-One Noisy Power Method

In contrast, our hierarchical procedure requires only a binary split at each internal node. We therefore specialize the noisy power method to the rank-one setting, where the iterate is a vector and the final split is determined from the sign pattern of the estimated dominant eigenvector of $A_{S}$ under the structural condition introduced before.

For a cluster *S*, let $x_{S}^{\left(0\right)}\in R^{\left|S\right|}$ satisfy ${∥x_{S}^{\left(0\right)}∥}_{2}=1$. At a depth *ℓ*, we run

(7) $$y_{S}^{\left(t\right)}=A_{S}x_{S}^{\left(t-1\right)}+z_{S}^{\left(t\right)},\;x_{S}^{\left(t\right)}=\frac{y_{S}^{\left(t\right)}}{∥y_{S}^{\left(t\right)}{∥}_{2}},\;t=1,\ldots ,N_{l},$$

where $z_{S}^{\left(t\right)}∼N\left(0,\sigma^{2}I_{\left|S\right|}\right)$ is a Gaussian perturbation. After $N_{l}$ iterations, the split is defined as follows:

(8) $$S^{+}=\left\{i\in S:x_{S,i}^{\left(N_{l}\right)}\geq 0\right\},\;S^{-}=S\setminus S^{+}.$$

Applying this procedure recursively from the root produces an estimated hierarchy $\hat{T}$. We next analyze the sensitivity of the multiplication $A_{S}x_{S}^{\left(t-1\right)}$ with respect to a change of a single edge as formalized in the following lemma.

**Lemma 1** 
(Local sensitivity of the rank-one power update)**.** *Let $A_{S}$ and $A_{S}^{\prime }$ be two adjacency matrices differing in one edge inside S. Then, for any unit vector $x\in R^{\left|S\right|}$, we have*

(9) $${∥A_{S}x-A_{S}^{\prime }x∥}_{2}\leq 1.$$

**Proof.**  If the changed edge is (i,j), then

$$A_{S}-A_{S}^{\prime }=\pm \left(e_{i}e_{j}^{⊤}+e_{j}e_{i}^{⊤}\right).$$

The above matrix has eigenvalues ±1 on the span of $\left\{e_{i},e_{j}\right\}$ and hence a spectral norm equal to 1. Therefore, we have

$${∥A_{S}x-A_{S}^{\prime }x∥}_{2}={∥\left(A_{S}-A_{S}^{\prime }\right)x∥}_{2}\leq {∥A_{S}-A_{S}^{\prime }∥}_{2}\cdot {∥x∥}_{2}\leq 1.$$

   □

Following the same Gaussian composition rule used in the noisy power method analysis for the flat problem, if the same noise scale is used across the $N_{l}$ iterations at a depth *ℓ*, then choosing

(10) $$\sigma_{l}=\varepsilon_{l}^{-1}\cdot \sqrt{4N_{l}\log\left(1/\delta_{l}\right)}$$

ensures that the entire trajectory at a depth *ℓ* is $\left(\varepsilon_{l},\delta_{l}\right)$-edge DP. To this end, we summarize the whole procedure of the noisy power method in Algorithm 1.

**Remark 1** 
(Remark on data-dependent iterates)**.** *Although Lemma 1 is stated for a fixed normalized vector x, the sensitivity bound is uniform over all x satisfying ${∥x∥}_{2}=1$. In Algorithm 1, the iterate $x_{S}^{\left(t-1\right)}$ is generally data dependent, as it is determined by the preceding noisy and normalized iterations. To account for this dependence, consider the mechanism at iteration t conditioned on the transcript of all preceding noisy outputs. Conditioned on this transcript, $x_{S}^{\left(t-1\right)}$ is fixed, and for any pair of edge-adjacent adjacency matrices $A_{S}$ and $A_{S}^{\prime }$, we have*

(11) $$\begin{array}{cc} {∥\left(A_{S}-A_{S}^{\prime }\right)x_{S}^{\left(t-1\right)}∥}_{2}\; & \leq {∥A_{S}-A_{S}^{\prime }∥}_{2}\cdot {∥x_{S}^{\left(t-1\right)}∥}_{2}\leq 1. \end{array}$$

*Hence, the conditional sensitivity of every iteration remains at most one, irrespective of how the current iterate was generated. Each iteration can therefore be analyzed as a conditional Gaussian mechanism, and the privacy guarantee of the complete noisy power trajectory follows from adaptive composition.*

**Algorithm 1** Recursive rank-one DP noisy power method.
**Require:** 
Adjacency matrix $A\in R^{n\times n}$, hierarchy depth *L*, iteration counts ${\left\{N_{l}\right\}}_{l=0}^{L-1}$, privacy budgets ${\left\{\left(\varepsilon_{l},\delta_{l}\right)\right\}}_{l=1}^{L}$**Ensure:** 
Estimated hierarchy $\hat{T}$  1:Initialize $C_{0}\leftarrow \left\{V\right\}$  2:**for** l=0,1,…,L−1 **do**  3:   Set $C_{l+1}\leftarrow \emptyset$  4:   **for** each cluster $S\in C_{l}$ **do**  5:     Form the induced adjacency matrix $A_{S}\in R^{\left|S|\times |S\right|}$  6:     Initialize $x_{S}^{\left(0\right)}\in R^{\left|S\right|}$ with ${∥x_{S}^{\left(0\right)}∥}_{2}=1$  7:     **for** $t=1,\ldots ,N_{l}$ **do**  8:        Sample $z_{S}^{\left(t\right)}∼N\left(0,\sigma_{l}^{2}I_{\left|S\right|}\right)$  9:        $y_{S}^{\left(t\right)}\leftarrow A_{S}x_{S}^{\left(t-1\right)}+z_{S}^{\left(t\right)}$10:        $x_{S}^{\left(t\right)}\leftarrow y_{S}^{\left(t\right)}/{∥y_{S}^{\left(t\right)}∥}_{2}$11:     **end for**12:     Define $S^{+},S^{-}$ according to (8)13:     Add $S^{+},S^{-}$ to $C_{l+1}$ and to $\hat{T}$14:   **end for**15:
**end for**
16:**return** $\hat{T}$


### 2.4. Auxiliary Lemma for Gaussian Concentration

The noisy-power utility analysis relies on standard concentration bounds for Gaussian projections and Gaussian norms. The following lemma is the local version needed for our recursive procedure.

**Lemma 2** 
(Gaussian concentration for local noisy power iterations)**.** *Let $u\in R^{m}$ be a unit vector, and let $g_{1},\ldots ,g_{N}\overset{i.i.d.}{∼}N\left(0,\sigma^{2}I_{m}\right)$. Then, for any η∈(0,1) with a probability of at least 1−η, we have*

(12) $$\begin{array}{cc} \max_{t\in [N]}\left|u^{⊤}g_{t}\right|\; & \leq \sigma \cdot \sqrt{2\log\left(2N/\eta \right)}, \end{array}$$

(13) $$\begin{array}{cc} \max_{t\in [N]}{∥g_{t}∥}_{2}\; & \leq \sigma \cdot \left(\sqrt{m}+\sqrt{2\log\left(2N/\eta \right)}\right). \end{array}$$

**Proof.**  For a fixed *t*, since u is a unit norm, $u^{⊤}g_{t}∼N\left(0,\sigma^{2}\right)$. Standard Gaussian tail bounds yield

$$\mathrm{Pr}\left(|u^{⊤}g_{t}|\geq \sigma r\right)\leq 2e^{-r^{2}/2}.$$

Similarly, we have

$$\mathrm{Pr}\left({∥g_{t}∥}_{2}\geq \sigma \cdot \left(\sqrt{m}+r\right)\right)\leq e^{-r^{2}/2}.$$

Applying a union bound over t=1,…,N and choosing $r=\sqrt{2\log(2N/\eta )}$ yields the result. □

### 2.5. Local Accuracy of the Sign Split

The next lemma converts the Euclidean vector error into sign-based split error.

**Lemma 3** 
(Split error bound)**.** *Let $x_{S}\in R^{m}$ be a unit vector. Then, we have*

(14) $${\mathrm{err}}_{S}\leq \frac{m}{4}\cdot \min_{s\in \{\pm 1\}}{∥x_{S}-su_{S}^{★}∥}_{2}^{2}.$$

*Moreover, if*

(15) $$\min_{s\in \{\pm 1\}}{∥x_{S}-su_{S}^{★}∥}_{\infty }<\frac{1}{\sqrt{m}},$$

*then ${\mathrm{err}}_{S}=0$.*

**Proof.**  Fix s∈{±1}. If node *i* is misclassified by the sign rule, then $x_{S,i}$ and $su_{S,i}^{★}$ have opposite signs. Since $|u_{S,i}^{★}|=1/\sqrt{m}$, we must have

$$|x_{S,i}-su_{S,i}^{★}|\geq \frac{1}{\sqrt{m}}.$$

Thus, every misclassified coordinate contributes at least 1/m to the squared Euclidean error, which implies

$${\mathrm{err}}_{S}\leq \frac{m}{4}\cdot {∥x_{S}-su_{S}^{★}∥}_{2}^{2}.$$

Minimizing over *s* proves Equation (14). If Equation (15) holds, then every coordinate preserves its sign, and thus the split is exact. □

### 2.6. Rank-One Noisy Power Method at a Hierarchy Node

**Theorem 1** 
(Rank-one noisy power method at node *S*)**.** *Fix a node S⊆V with m=|S|, and let $u_{S}^{★}$ denote the leading unit eigenvector of $A_{S}$. Suppose that the initialization satisfies*

$$|\langle x_{S}^{\left(0\right)},u_{S}^{★}\rangle |\geq \gamma_{S}>0.$$

*Choose*

(16) $$\sigma_{l}=\varepsilon_{l}^{-1}\cdot \sqrt{4N_{l}\log\left(1/\delta_{l}\right)}.$$

*Then, the rank-one noisy power iteration (Equation* (7)*) at node S is $\left(\varepsilon_{l},\delta_{l}\right)$-edge DP. Moreover, after*

(17) $$N_{l}=O\;\left(\frac{\lambda_{1}\left(A_{S}\right)}{\Delta_{S}}\cdot \log m\right)$$

*iterations, we have for any η∈(0,1)*

(18) $$\min_{s\in \{\pm 1\}}{∥x_{S}^{\left(N_{l}\right)}-su_{S}^{★}∥}_{2}\leq \frac{\sqrt{2}\;\sigma_{l}}{\Delta_{S}}\cdot \left(\sqrt{m}+\sqrt{2\log\left(2N_{l}/\eta \right)}\right)$$

*with a probability of at least 1−η.*

**Proof.**  The privacy claim follows from Lemma 1 together with the Gaussian composition argument used in the classical noisy power analysis. The $N_{l}$-step composition of Gaussian mechanisms with unit sensitivity yields $\left(\varepsilon_{l},\delta_{l}\right)$-edge privacy under the choice (16). For utility, the standard noisy power method analysis yields a bound on the residual

$${∥\left(I-u_{S}^{★}{\left(u_{S}^{★}\right)}^{⊤}\right)\cdot x_{S}^{\left(N_{l}\right)}∥}_{2}\leq \frac{\sigma_{l}}{\Delta_{S}}\cdot \left(\sqrt{m}+\sqrt{2\log\left(2N_{l}/\eta \right)}\right)$$

after $N_{l}=O\left(\left(\lambda_{1}\left(A_{S}\right)/\Delta_{S}\right)\cdot \log m\right)$ iterations. This is the rank-one specialization of the corresponding subspace estimate in the flat noisy power method. Finally, since both $x_{S}^{\left(N_{l}\right)}$ and $u_{S}^{★}$ are unit vectors, we have

$$\min_{s\in \{\pm 1\}}∥x_{S}^{\left(N_{l}\right)}-su_{S}^{★}{∥}_{2}\leq \sqrt{2}\;\cdot {∥\left(I-u_{S}^{★}{\left(u_{S}^{★}\right)}^{⊤}\right)\cdot x_{S}^{\left(N_{l}\right)}∥}_{2},$$

which gives (18). □

The analysis of the noisy power iteration follows along similar lines to our previous work [19], where the initialization alignment, eigenvalue ordering and eigengap conditions, and the effect of the Gaussian perturbation relative to the spectral signal are analyzed in more detail.

### 2.7. Depth-Adaptive Iteration Design

The preceding result shows that the utility of the local noisy power method depends on the number of iterations $N_{l}$. Increasing $N_{l}$ improves the deterministic contraction of the power method, but it also increases the noise scale $\sigma_{l}\left(N_{l}\right)$ required by privacy composition. We therefore choose $N_{l}$ adaptively by balancing these two effects. Let $u_{S}^{★}$ denote the leading unit eigenvector of $A_{S}$, and suppose that the initialization satisfies

$$\gamma_{S}:=\left|\langle x_{S}^{\left(0\right)},u_{S}^{★}\rangle \right|>0.$$

In the absence of noise, the normalized power method contracts toward $u_{S}^{★}$ as follows:

(19) $$\min_{s\in \{\pm 1\}}{∥x_{S}^{\left(t\right)}-su_{S}^{★}∥}_{2}\leq \sqrt{2}\;\cdot \frac{\rho_{S}^{t}}{\gamma_{S}},$$

where $\rho_{S}$ is the contraction ratio. Thus, the deterministic bias decays exponentially with *t*.

On the other hand, by the privacy calibration in Equation (10), the Gaussian noise scale after *t* iterations at a depth *ℓ* is

$$\sigma_{l}\left(t\right)=\frac{1}{\varepsilon_{l}}\cdot \sqrt{4t\log\left(\frac{1}{\delta_{l}}\right)}.$$

Together with the local noisy power bound in Theorem 1, this induces a stochastic error level of the order

(20) $$\frac{\sigma_{l}\left(t\right)}{\Delta_{S}}\cdot \left(\sqrt{m}+\sqrt{2\log\left(\frac{2t}{\eta }\right)}\right).$$

Ignoring the lower-order logarithmic term in Equation (20), the local error after *t* iterations is therefore controlled by

(21) $${\mathrm{err}}_{S}\left(t\right)\;≲\;\underset{\mathrm{deterministic}\;\mathrm{contraction}}{\underbrace{\frac{\rho_{S}^{t}}{\gamma_{S}}}}+\underset{\mathrm{privacy}\text{-}\mathrm{induced}\;\mathrm{floor}}{\underbrace{\frac{\sigma_{l}\left(t\right)\sqrt{m}}{\Delta_{S}}}}.$$

This motivates the stopping rule

(22) $$N_{l}:=\min\left\{t\in N:\frac{\rho_{S}^{t}}{\gamma_{S}}\leq c\;\frac{\sigma_{l}\left(t\right)\sqrt{m}}{\Delta_{S}}\right\},$$

where c>0 is an absolute numerical constant. This rule stops the noisy power iteration once the residual deterministic bias reaches the privacy-induced noise floor. Substituting the expression for $\sigma_{l}\left(t\right)$ into Equation (22) gives the approximate balance

$$\frac{\rho_{S}^{t}}{\gamma_{S}}≍\frac{\sqrt{t\;m\log(1/\delta_{l})}}{\varepsilon_{l}\Delta_{S}}.$$

Let

$$a_{S}:=\log\left(1/\rho_{S}\right),\;K_{S}:=\frac{\gamma_{S}\sqrt{m\log(1/\delta_{l})}}{\varepsilon_{l}\Delta_{S}}.$$

Then, the balance can be written as

$$e^{-a_{S}t}≍K_{S}\sqrt{t}.$$

Squaring both sides and solving using the Lambert *W* function gives the continuous approximation

(23) $$N_{l}\approx \frac{1}{2a_{S}}\cdot W\;\left(\frac{2a_{S}}{K_{S}^{2}}\right).$$

Equivalently, at the leading logarithmic order, we have

(24) $$N_{l}=\Theta \;\left(\frac{1}{\log(1/\rho_{S})}\cdot \log\left(\frac{\gamma_{S}\varepsilon_{l}\Delta_{S}}{\sqrt{m\log(1/\delta_{l})}}\right)\right),$$

up to lower-order logarithmic corrections.

### 2.8. Exact Recovery of the Hierarchy

Since the algorithm proceeds recursively, the cleanest proof strategy is inductive: if each local split is recovered exactly, then the algorithm continues on the correct descendant clusters.

**Theorem 2** 
(Exact recovery of the hierarchy)**.** *Suppose that for every internal node S⊆V at a depth ℓ, we have*

(25) $$\frac{\sqrt{2}\;\sigma_{l}}{\Delta_{S}}\left(\sqrt{m}+\sqrt{2\log\left(2N_{l}/\eta \right)}\right)<\frac{1}{\sqrt{m}}.$$

*Then, the recursive rank-one noisy power method recovers every split correctly and hence recovers the entire hierarchy $T^{★}$, with a probability of at least 1−η.*

**Proof.**  Under Theorem 1, the condition in Equation (25) implies that for every internal node *S*, we have

$$\min_{s\in \{\pm 1\}}{∥x_{S}^{\left(N_{l}\right)}-su_{S}^{★}∥}_{2}<\frac{1}{\sqrt{m}}$$

with a probability of at least 1−η. Since ${∥\cdot ∥}_{\infty }\leq {∥\cdot ∥}_{2}$, we also have

$$\min_{s\in \{\pm 1\}}{∥x_{S}^{\left(N_{l}\right)}-su_{S}^{★}∥}_{\infty }<\frac{1}{\sqrt{m}}.$$

Lemma 3 then implies that the sign split at node *S* is exact. At the root, this yields the correct split of *V*. When conditioning on correctness up to a depth l−1, the algorithm at a depth *ℓ* operates on the true clusters in $T^{★}$, and thus the same argument applies recursively. Therefore, every internal node is split correctly, and the full hierarchy is recovered. □

### 2.9. Tighter Privacy Analysis for Noisy Power Iteration

To further optimize the privacy-utility trade-offs for the noisy power iteration for numerical implementation, we consider a tighter privacy analysis via so-called dominating pairs of distributions [39]. The noisy power iteration algorithm with *N* steps can be see as an adaptive composition of the form

(26) $$\begin{array}{cc} M^{\left(N\right)}\left(G\right) & =\left(M_{1}\left(G\right),M_{2}\left(M_{1}\left(G\right),G\right),\cdots ,M_{N}\left(M_{1}\left(G\right),\cdots ,M_{N-1}\left(G\right),G\right)\right). \end{array}$$

We obtain accurate (ϵ,δ)-edge DP guarantees for adaptive compositions by leveraging dominating pairs of distributions, as introduced in [39]. Due to the scaling of the noise in Algorithm 1, the privacy loss at each step is dominated by that of the Gaussian mechanism with a sensitivity of one and a noise standard deviation σ. Accordingly, the dominating pair of distributions for each step is given by (P,Q), where $P=N(1,\sigma^{2})$ and $Q=N(0,\sigma^{2})$. As is shown in [40], the corresponding privacy loss random variable (PLRV) $\omega_{P/Q}$ is then distributed as follows:

$$\omega_{P/Q}∼N\left(\frac{1}{2\sigma^{2}},\frac{1}{\sigma^{2}}\right).$$

When applying standard composition results for adaptive compositions [39,41], it follows that the PLRV of an *N*-fold adaptive composition of Algorithm 1 is given by a sum of *N* independent random variables, each distributed as $\omega_{P/Q}$. Thus, the resulting PLRV for the *N*-fold adaptive composition of Algorithm 1 is distributed as $N\left(\frac{N}{2\sigma^{2}},\frac{N}{\sigma^{2}}\right)$, and the resulting (ϵ,δ)-DP guarantee corresponds to that of the Gaussian mechanism with a sensitivity of one and a noise parameter $\sigma /\sqrt{N}$. The analytical expression from [42] (Thm. 8) then yields the following bound.

**Lemma 4.** 
*The proposed algorithm is $\left(\epsilon ,\delta (\epsilon )\right)$- edge DP for*

$$\delta \left(\epsilon \right)=\Phi \left(-\frac{\epsilon \sigma }{\sqrt{T}}+\frac{\sqrt{N}}{2\sigma }\right)-e^{\epsilon }\Phi \left(-\frac{\epsilon \sigma }{\sqrt{N}}-\frac{\sqrt{N}}{2\sigma }\right),$$

*where* Φ *denotes the CDF of the standard univariate Gaussian distribution.*

To compute ϵ as a function σ and δ or σ as a function of ϵ and δ, the expression of Lemma 4 can be inverted using, for example, the bisection method.

**Remark 2** 
(Privacy of the complete hierarchy)**.** *The privacy guarantees above are stated for the noisy power mechanism applied at a given recursive level. We now clarify the privacy guarantee for the complete released hierarchy. At any fixed depth ℓ, conditioned on the hierarchy released up to a depth l−1, the current clusters form a disjoint partition of the vertex set. Consequently, each edge of the input graph belongs to at most one of the induced subgraphs processed at a depth ℓ. Therefore, the mechanisms applied to the different clusters at the same depth can be handled using parallel composition. In particular, if every mechanism at a depth ℓ satisfies $\left(\epsilon_{l},\delta_{l}\right)$-edge DP, then the joint release at that depth also satisfies $\left(\epsilon_{l},\delta_{l}\right)$-edge DP; the privacy parameters are not summed over the clusters at the same depth.*

*Across different depths, however, the same edge may be accessed repeatedly along the root-to-leaf trajectory containing its endpoints. Hence, parallel composition does not apply across recursive levels. Instead, the privacy loss of the complete hierarchy is obtained via adaptive composition of the depth-dependent mechanisms:*

$$\begin{array}{c} \left(\epsilon_{1},\delta_{1}\right),\;\left(\epsilon_{2},\delta_{2}\right),\;\ldots ,\;\left(\epsilon_{L},\delta_{L}\right). \end{array}$$

*A direct application of basic composition gives the conservative guarantee*

(27) $$\begin{array}{cc} \epsilon_{\mathrm{tot}}\; & \leq \sum_{l=1}^{L}\epsilon_{l},\\ \delta_{\mathrm{tot}}\; & \leq \sum_{l=1}^{L}\delta_{l}. \end{array}$$

*A tighter guarantee can be obtained by allowing an additional failure probability $\delta^{\prime }>0$ and applying advanced heterogeneous composition [13]. In particular, the complete hierarchy satisfies $\left(\epsilon_{\mathrm{tot}},\delta_{\mathrm{tot}}\right)$-edge DP with*

(28) $$\begin{array}{cc} \epsilon_{\mathrm{tot}}\; & \leq \sqrt{2\log\left(\frac{1}{\delta^{\prime }}\right)\sum_{l=1}^{L}\epsilon_{l}^{2}}+\sum_{l=1}^{L}\epsilon_{l}\left(e^{\epsilon_{l}}-1\right), \end{array}$$

(29) $$\begin{array}{cc} \delta_{\mathrm{tot}}\; & \leq \delta^{\prime }+\sum_{l=1}^{L}\delta_{l}. \end{array}$$

*Thus, the overall privacy leakage depends on the depth-wise privacy budgets rather than on the total number of clusters in the hierarchy. For the common choice of equal depth-wise budgets $\epsilon_{l}=\epsilon_{0}$ and $\delta_{l}=\delta_{0}$ for all ℓ, the above simplifies to*

(30) $$\begin{array}{cc} \epsilon_{\mathrm{tot}}\; & \leq \epsilon_{0}\sqrt{2L\log\left(\frac{1}{\delta^{\prime }}\right)}+L\epsilon_{0}\left(e^{\epsilon_{0}}-1\right), \end{array}$$

(31) $$\begin{array}{cc} \delta_{\mathrm{tot}}\; & \leq L\delta_{0}+\delta^{\prime }. \end{array}$$

*For small $\epsilon_{0}$ values, since $e^{\epsilon_{0}}-1\approx \epsilon_{0}$, this gives the useful scaling*

(32) $$\begin{array}{c} \epsilon_{\mathrm{tot}}≲\epsilon_{0}\sqrt{2L\log\left(\frac{1}{\delta^{\prime }}\right)}+L\epsilon_{0}^{2}, \end{array}$$

*which can be substantially smaller than the basic-composition bound $L\epsilon_{0}$.*

## 3. Experimental Results

In this section, we evaluate the performance of the proposed private algorithm on both synthetic and real-world graph datasets. In each experiment, we report the clustering error rate over a range of ε values selected on a logarithmically spaced grid. All results were averaged over 20 independent trials.

We first considered a balanced hierarchical stochastic block model (HSBM) in which the *n* vertices were organized according to a binary tree and the leaves corresponded to the ground-truth primitive communities. Let *k* denote the number of primitive communities, i.e., the number of leaves in the underlying hierarchy. At each internal node, the associated vertex set was recursively partitioned into two sub-groups, thereby inducing a sequence of binary splits from coarse to fine scales. The edge probabilities were chosen hierarchically so that vertices sharing a deeper common ancestor were more likely to be connected than vertices that separated higher in the tree.

**Figure 2:**
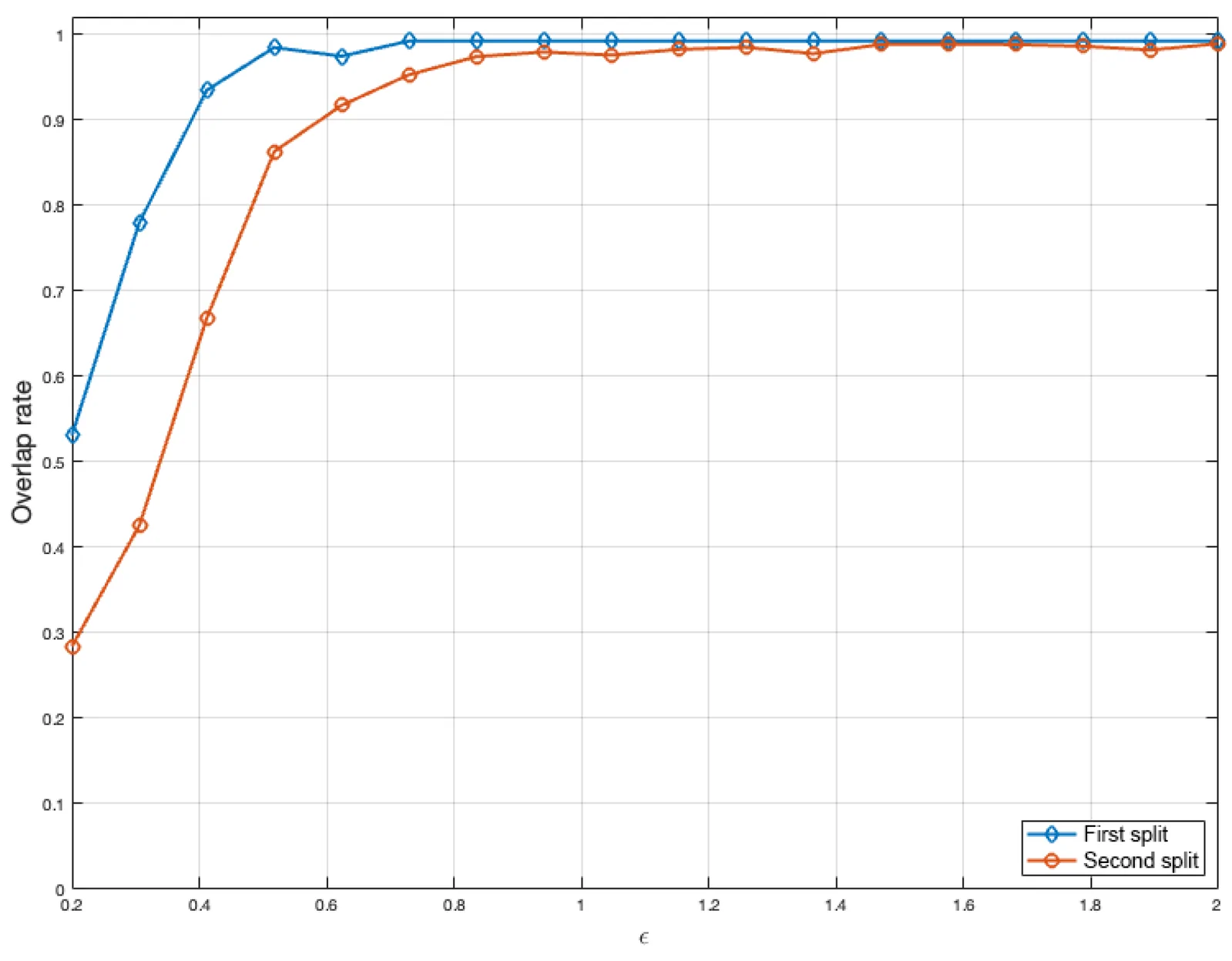
First-split overlap (clustering accuracy) as a function of the privacy budget ε under a balanced HSBM, where k=4, $p_{0}=0.02$, $p_{1}=0.08$, and $p_{2}=0.2$. As ε increased (weaker privacy), the accuracy improved due to reduced noise in the private power iterations.

As a first example, we considered an HSBM with k=4 primitive communities, each with a size of 200. The top-level split separated the four communities into two groups, while the second level further refined each group into its two constituent primitive communities. Edges were generated independently with a probability $p_{2}=0.2$ within each primitive community, a probability $p_{1}=0.08$ between communities that shared the same parent in the hierarchy, and a probability $p_{0}=0.02$ between communities that separated at the top level. Figure 2 plots the overlap rate versus the privacy budget ε for the proposed noisy power method for both the first and second splits. As expected, the first split was recovered more accurately, since it corresponded to the strongest level of separation in the hierarchy.

**Figure 3:**
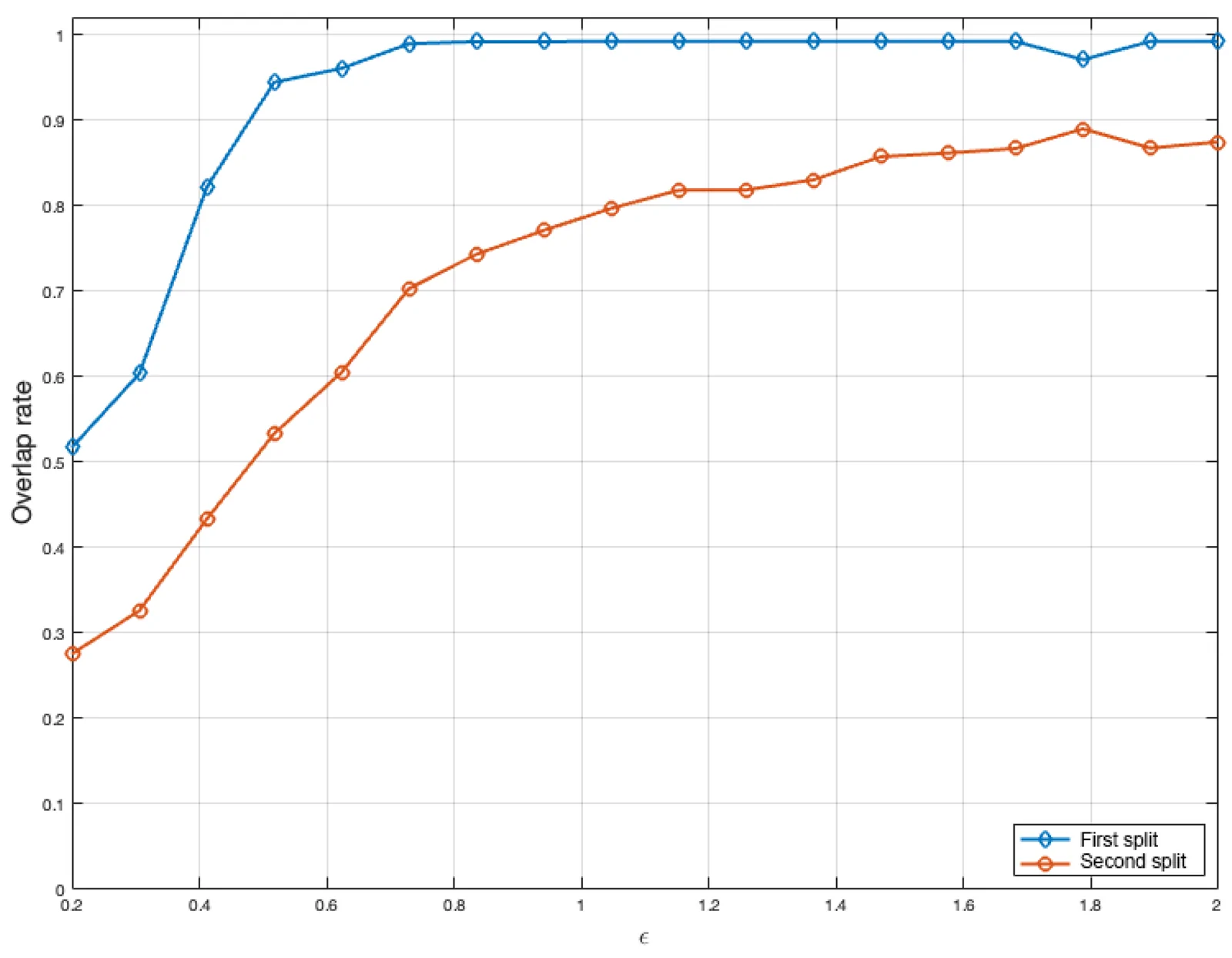
First-split overlap (clustering accuracy) as a function of the privacy budget ε under a balanced HSBM, where k=4, $p_{0}=0.02$, $p_{1}=0.08$, and $p_{2}=0.15$.

**Impact of connectivity.** To examine a more challenging regime, we also considered less-separated probability configurations (where $p_{2}=0.15$) in which the gap between within-group and across-group connectivity was reduced. As shown in Figure 3, the second split became substantially harder to recover, even when the first split remained relatively stable. This behavior reflects the fact that the local spectral signal became weaker deeper in the hierarchy.

These results highlight a key feature of hierarchical graph clustering under privacy: the privacy-utility trade-off becomes increasingly delicate as one moves down the tree. Coarse, upper-level splits were typically separated well and therefore robust to privacy noise, whereas finer splits relied on weaker local structure and were correspondingly more sensitive to perturbation. Even when the same privacy level was used at each split, deeper levels of the hierarchy exhibited noticeably larger degradation.

**Figure 4:**
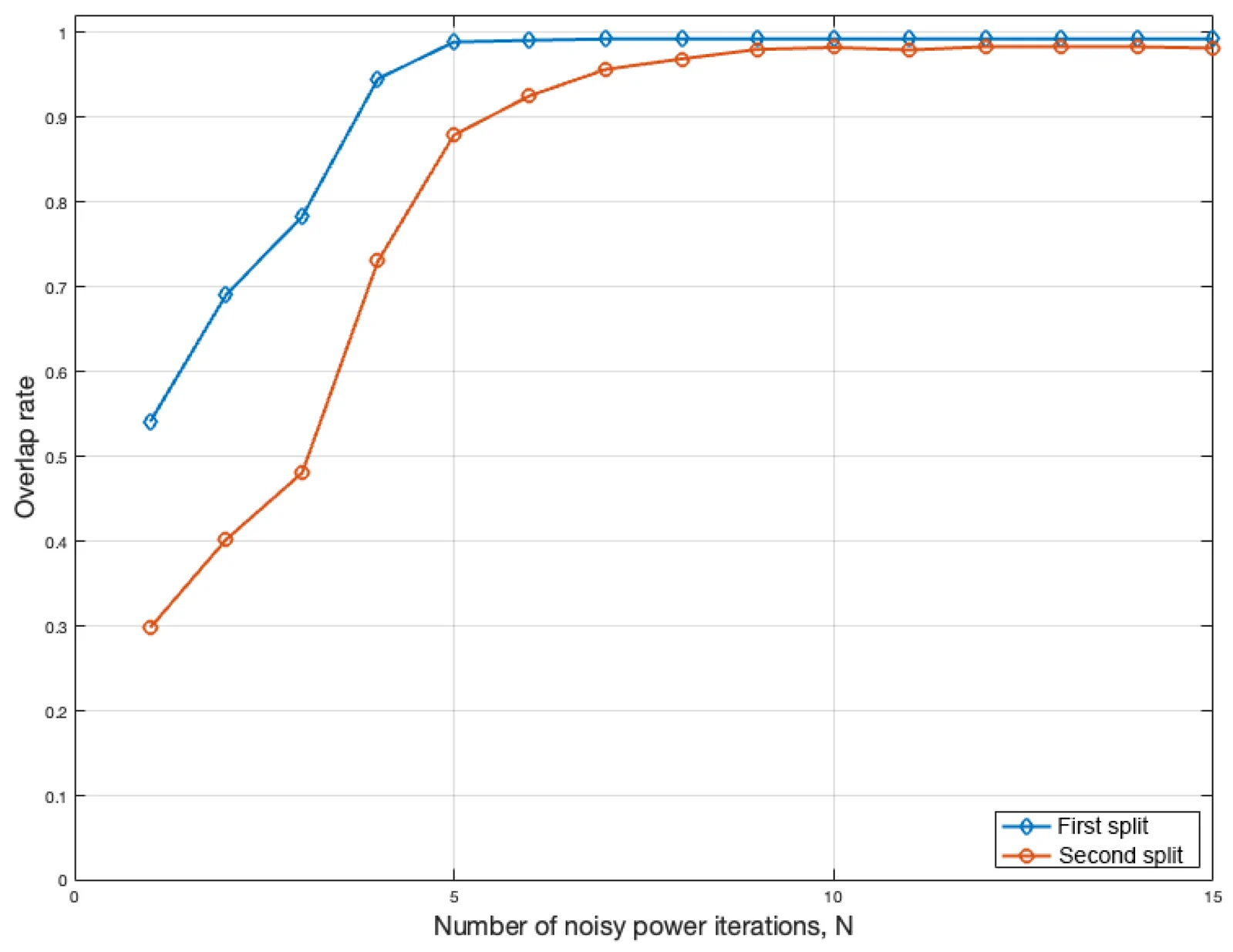
Overlap rate versus the number of noisy power iterations *N* under a fixed privacy budget ε=1. We considered an HSBM with k=4 communities, each with a size of 400, and edge probabilities $p_{2}=0.20$, $p_{1}=0.08$, and $p_{0}=0.02$.

**Impact of noisy power iterations.** We next examined the impact of the number of noisy power iterations on the clustering performance under a fixed privacy budget. Figure 4 shows the overlap rate for both the first and second splits as a function of the iteration count *N*. For small values of *N*, the performance was limited due to insufficient spectral convergence, as the power method had not yet aligned with the leading eigenvector. As *N* increased, the overlap improved, reflecting better estimation of the underlying partition structure. However, beyond a certain point, the performance saturated and may have slightly degraded, which is consistent with the accumulation of privacy noise across iterations. Overall, the results indicate that a small number of iterations is sufficient to achieve near-optimal performance, highlighting the trade-off between convergence and noise in the differentially private setting. Moreover, the first split consistently achieved greater accuracy than the second split, as it operated on a larger graph with a stronger effective signal-to-noise ratio.

**Figure 5:**
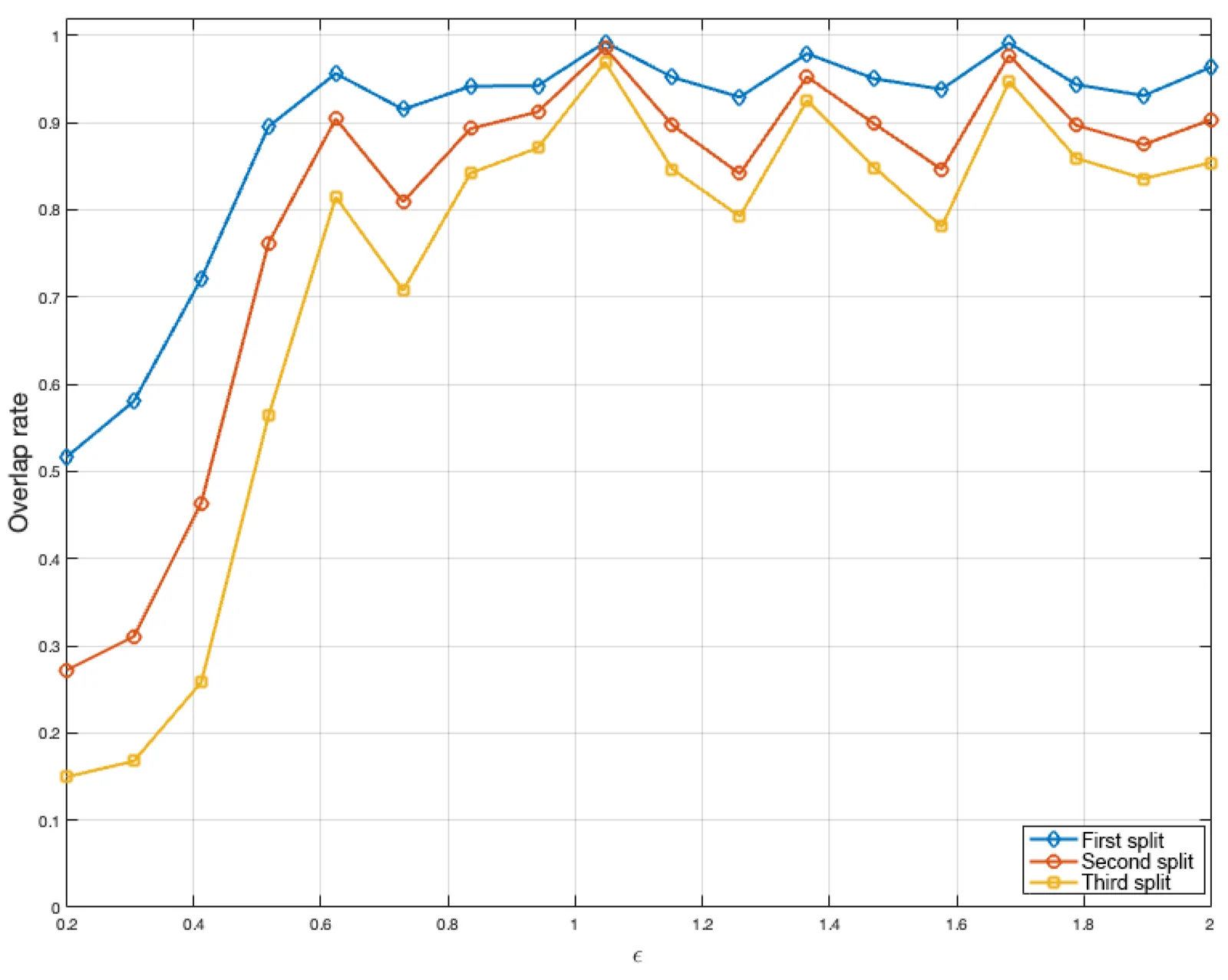
Overlap rate versus privacy budget ε for an HSBM with k=8 communities, each with a size of 200. The edge probabilities were $p_{3}=0.20$ (within-community), $p_{2}=0.08$ (same parent), $p_{1}=0.04$ (same grandparent), and $p_{0}=0.02$ (across top-level partitions). The recursive noisy power method was applied for three levels of splitting using N=8 iterations per level. The first split remained robust across privacy levels, while deeper splits exhibited increased sensitivity to noise due to error propagation and reduced effective signal strength.

**Impact of the number of communities.** Figure 5 illustrates the overlap rate of the recursive noisy power method as a function of the privacy budget ε for a hierarchical stochastic block model with k=8 communities. Each curve corresponds to a different level of the hierarchy, namely the first, second, and third binary splits. As expected, the overlap rate improved as ε increased, reflecting the reduction in privacy-induced noise. Moreover, the first split consistently achieved the highest accuracy across all privacy regimes, as it corresponded to the coarsest partition with the largest effective signal strength. In contrast, the second and third splits exhibited progressively lower overlap rates, highlighting the cumulative impact of noise and the reduced separation between sub-communities at deeper levels of the hierarchy.

**Figure 6:**
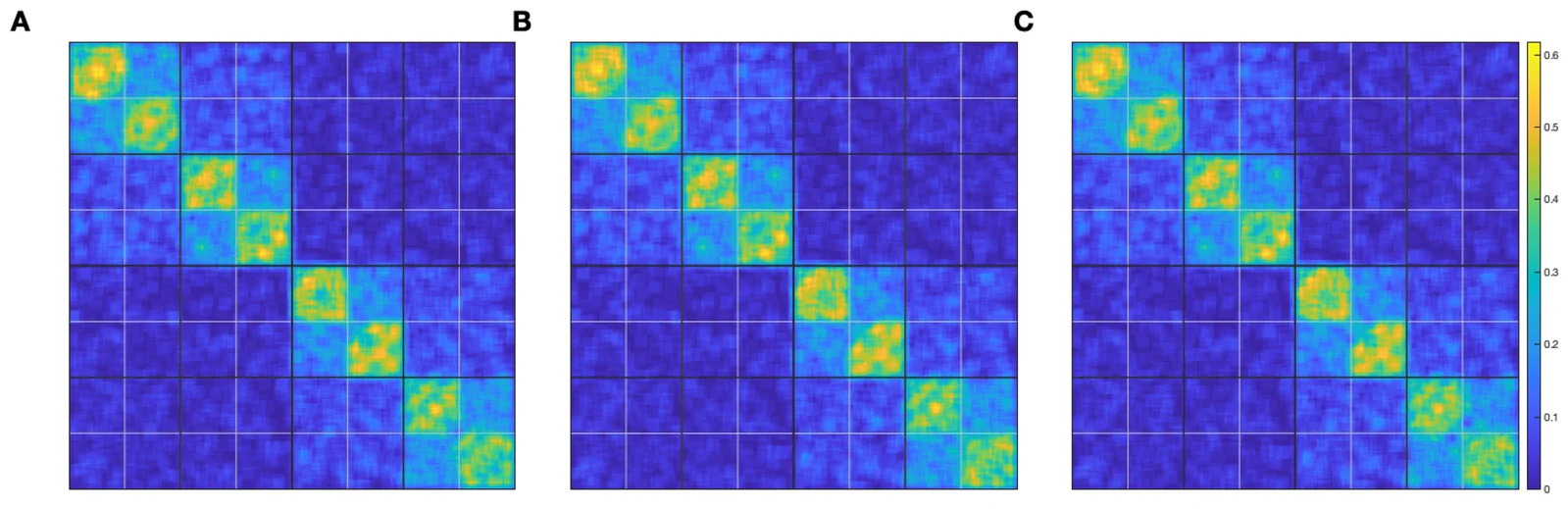
Impact of privacy on hierarchical community recovery under a balanced HSBM. The graph consists of $n=kn_{0}=320$ vertices arranged according to a binary tree of a depth L=3, with $k=2^{L}=8$ primitive communities of equal size $n_{0}=40$. Edges were generated independently with a probability $p_{d}$ determined by the depth d∈{0,1,2,3} of the lowest common ancestor, where $\left(p_{0},p_{1},p_{2},p_{3}\right)=\left(0.03,\;0.08,\;0.18,\;0.42\right)$. (**A**) Ground-truth ordering. (**B**) Reordering induced by the proposed private recursive noisy power method with ϵ=1. (**C**) Reordering under stronger privacy, ϵ=0.5. Each recursive split used T=25 noisy power iterations with Gaussian perturbations. Stronger privacy resulted in larger perturbations and visibly degraded recovery of the multiscale hierarchical structure.

To further illustrate the effect of privacy on hierarchical community recovery, we considered two balanced hierarchical random graph examples. In the first example, shown in Figure 6, the graph has a depth L=3, corresponding to $k=2^{3}=8$ primitive communities. The left panel shows the smoothed adjacency matrix under the ground-truth ordering, where the nested block structure induced by the hierarchy is clearly visible. The middle and right panels show the same adjacency matrix reordered according to the hierarchy estimated by the proposed private recursive noisy power method under privacy budgets ϵ=1 and ϵ=0.5, respectively. As privacy became stronger, the recovered multiscale block structure became less distinct due to the larger perturbation introduced during recursive private spectral splitting.

**Figure 7:**
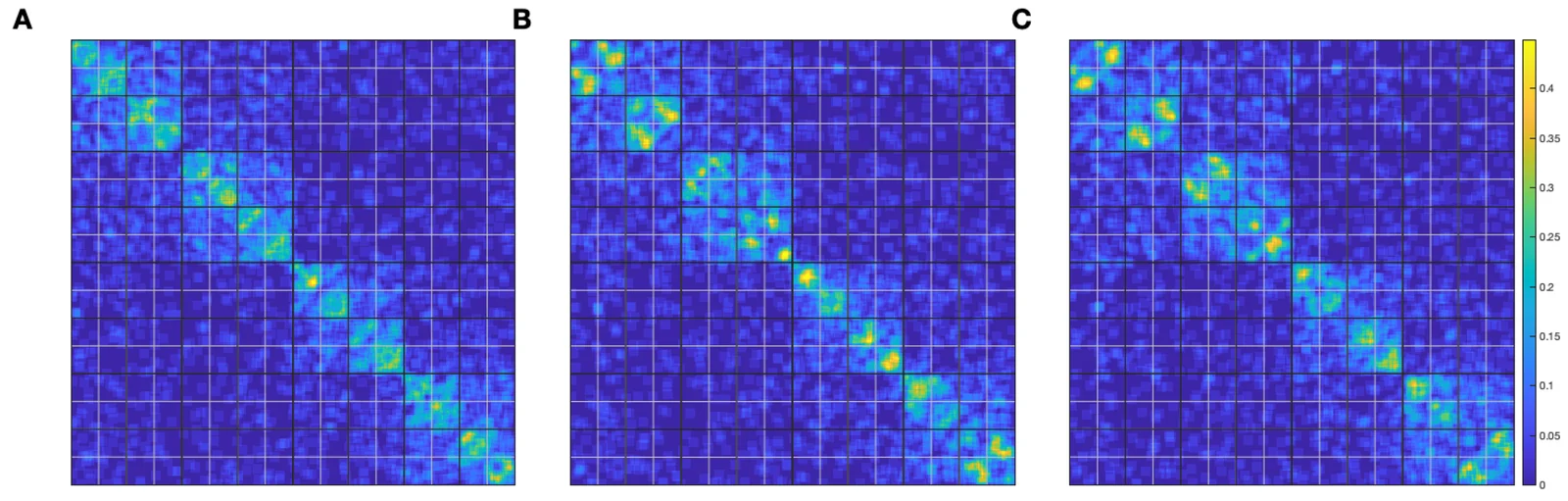
Impact of privacy on hierarchical community recovery for a deeper hierarchical random graph. The graph consists of $n=kn_{0}=320$ vertices arranged according to a balanced binary hierarchy of a depth L=4, with $k=2^{L}=16$ primitive communities of equal size $n_{0}=20$. Edges were generated independently according to a hierarchical random graph model, where the connection probability between two vertices depends on the depth of their lowest common ancestor, with $\left(p_{0},p_{1},p_{2},p_{3},p_{4}\right)=\left(0.015,\;0.03,\;0.06,\;0.12,\;0.24\right)$. (**A**) Ground-truth ordering. (**B**) Reordering induced by the proposed private recursive noisy power method with ϵ=1. (**C**) Reordering under stronger privacy, ϵ=0.5.

Figure 7 presents a deeper hierarchical example with a depth L=4, corresponding to $k=2^{4}=16$ primitive communities. Although the total graph size remained the same, increasing the depth produced a finer multiscale organization with more nested communities. The ground-truth ordering again revealed the hierarchical structure, while the private reconstructions demonstrated that recovering deeper hierarchical levels becomes increasingly challenging under privacy constraints. In particular, under the smaller privacy budget ϵ=0.5, the finest-scale blocks were more strongly distorted, illustrating the growing difficulty of accurate hierarchical recovery as both the hierarchy depth and privacy level increase.

### 3.1. Real-World Network

We next compare recursive spectral bi-clustering based on the adjacency matrix A on a real-world network.

The Football dataset [43] represents a network of American college football teams during the fall 2000 regular season. Nodes correspond to teams, and edges indicate games played between pairs of teams. The teams are organized into conferences, each typically containing between 8 and 12 teams, which we treated as the ground-truth primitive communities. We again applied the recursive bi-partitioning algorithm based on the description of Algorithm 1 to construct a hierarchy. In this case, we obtained a balanced hierarchy with a depth of three without imposing an explicit stopping rule. Figure 6A–C depicts the first three levels of the inferred hierarchy, corresponding to two, four, and eight clusters, respectively. Each level achieved a high completeness score, indicating that the recovered hierarchy was well aligned with the underlying community structure.

**Figure 8:**
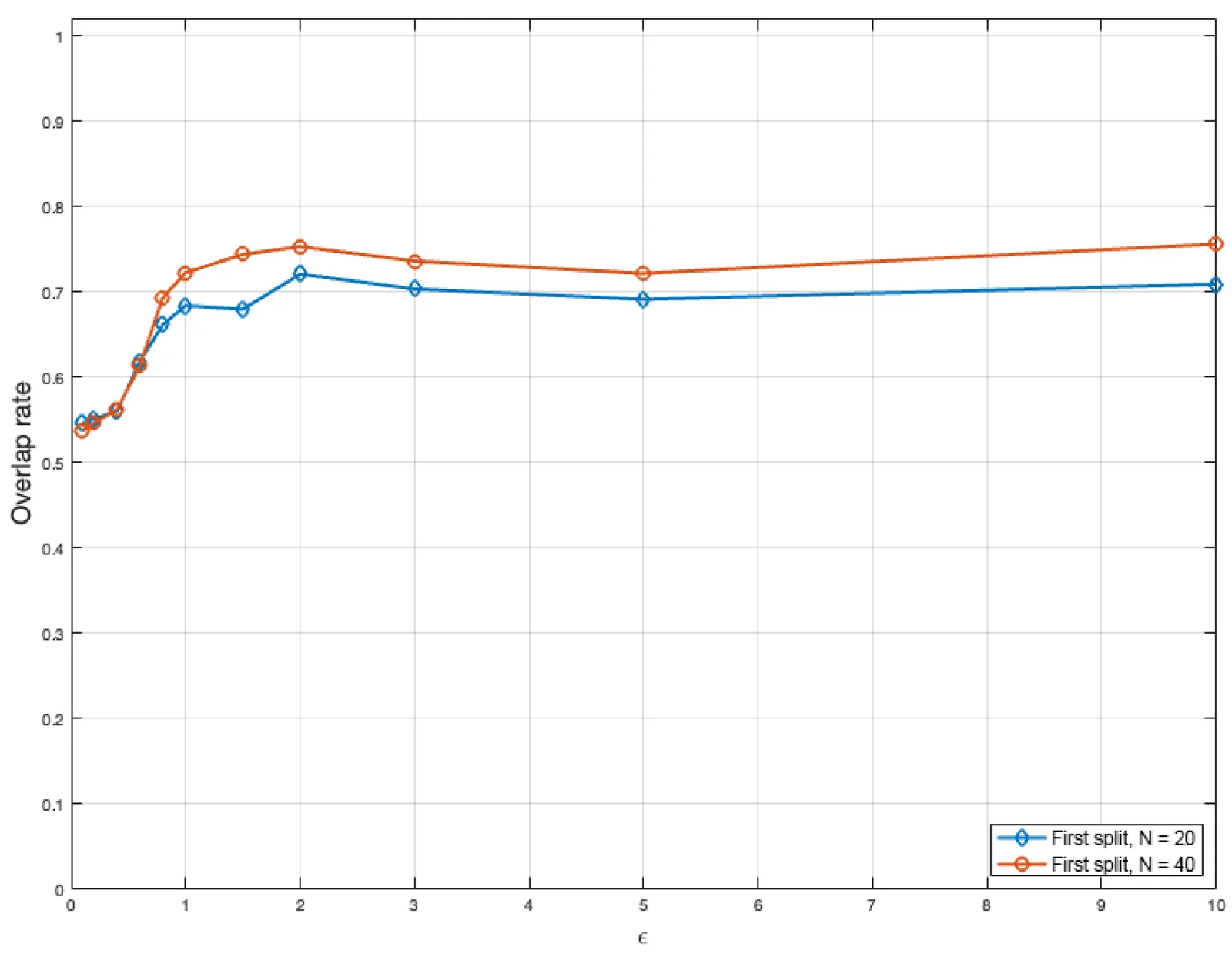
First-split overlap rate versus the privacy budget ϵ on the football network for N=20 and N=40 noisy power iterations.

Figure 8 reports the first-split overlap rate on the football network versus the privacy budget ϵ for two choices in the number of noisy power iterations, N=20 and N=40. The overlap tended to improve as ϵ increased, which is consistent with the fact that a larger privacy budget reduces the magnitude of the injected perturbation. The figure also shows the impact of the iteration count on the recovered partition; increasing *N* can enhance the spectral separation, but the improvement may be moderated by the cumulative effect of privacy noise over repeated iterations.

We emphasize that the imposed depth-three binary hierarchy is an algorithmic construction used to evaluate the recursive spectral procedure and should not be interpreted as the ground-truth conference hierarchy of the Football network, which is neither inherently binary nor balanced.

### 3.2. LLM-Inspired Semantic Graphs

As an LLM-motivated example, we constructed a similarity graph whose nodes corresponded to short text snippets related to four representative topics: watermarking, privacy, retrieval-augmented generation (RAG), and efficient inference. Each sentence was converted into a simple bag-of-words representation, reweighted using an inverse document frequency factor, and then normalized. We connected two nodes whenever the cosine similarity between their feature vectors exceeded a prescribed threshold (τ=0.2), thereby forming an undirected semantic similarity graph.

The resulting graph exhibits a natural hierarchical organization. At the coarse level, the sentences split into theory-oriented topics, namely watermarking and privacy, versus systems-oriented topics, namely RAG and efficient inference. At the finer level, each branch further separated into its constituent topics. This example illustrates how the proposed recursive private spectral clustering framework can be used beyond classical network data, organizing semantically related LLM-generated or LLM-related text samples through a graph-based representation.

**Figure 9:**
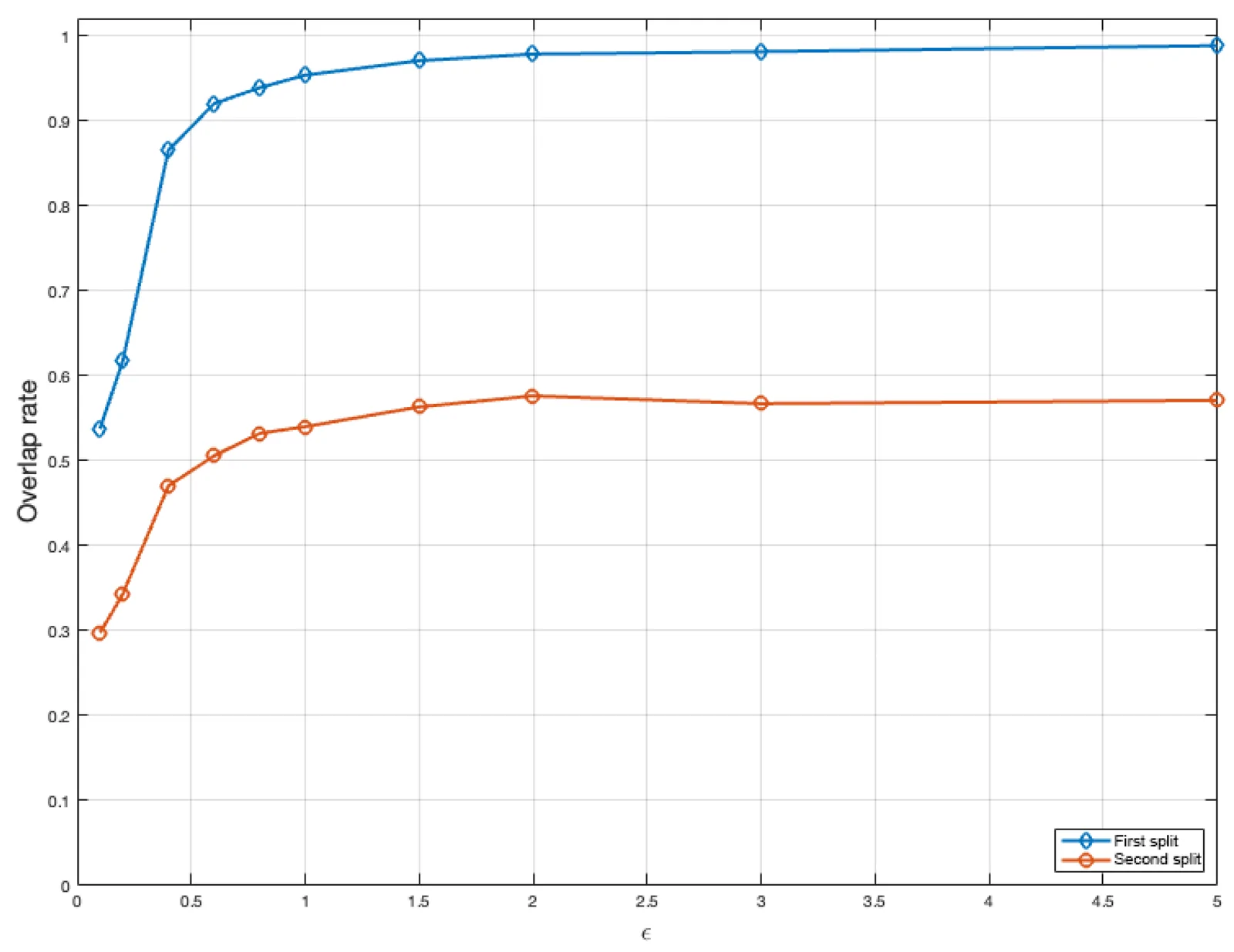
Overlap rate versus ε on the LLM sentence similarity graph for N=20 noisy power iterations.

Figure 9 shows the overlap rate as a function of the privacy budget ε on the LLM-inspired sentence similarity graph. The graph was constructed from topic-specific sentences using a TF-IDF representation and cosine similarity. As ϵ increased, the performance improved due to reduced perturbation in the noisy power iterations. We note that more sophisticated graph constructions based on contextual embeddings from modern language models could further enhance the quality of the similarity graph, as such embeddings are known to exhibit rich geometric structures, including clusters and low-dimensional manifolds [44]. The results demonstrate that the proposed method can effectively recover both coarse and fine semantic structure in LLM-related text data.

## 4. Conclusions and Future Work

In this paper, we studied the problem of hierarchical spectral clustering under edge differential privacy. We developed a recursive spectral framework based on a rank-one noisy power method applied to adjacency matrices, where carefully calibrated Gaussian noise is injected at each iteration to ensure (ε,δ)-edge DP through cumulative privacy accounting across both recursive iterations and hierarchy levels We provided a non-asymptotic analysis of the resulting noisy iterations, characterizing the trade-offs between privacy, accuracy, and computational efficiency. In particular, we quantified how the noise variance, iteration depth, and spectral properties jointly affect eigenvector estimation and downstream clustering performance. Our results further captured the propagation of errors across the hierarchy and established conditions for consistent recovery of the underlying multi-scale community structure. Empirical evaluations on synthetic and real-world networks validated our theoretical findings and demonstrated that the proposed approach achieves strong clustering performance under meaningful privacy budgets. An important direction for future work is to extend this framework to *attributed graphs*, where node features introduce additional sources of sensitivity, as well as to explore adaptive privacy budget allocation strategies across hierarchy levels to further improve utility.

## Figures and Tables

**Table 1:** Comparison with Imola et al. [31]. While both works consider edge-differentially private hierarchical clustering, they differ in their algorithmic formulation, utility objectives, and theoretical emphasis.

Aspect	Imola et al. [31]	This Work
Primary problem	Private hierarchical clustering formulated primarily through approximation of Dasgupta’s hierarchical clustering objective	Private hierarchical spectral clustering through recursive spectral partitioning
Core mechanism	Private cut sparsification or exponential mechanism for general graphs; private community recovery followed by noisy block-level hierarchical clustering for HSBMs	Gaussian-perturbed power iteration applied recursively to induced adjacency matrices
Hierarchy construction	In the HSBM setting, privately recovered blocks are combined using noisy inter-block similarities and a linkage-like merging procedure	Top-down recursive binary partitioning based on private spectral estimates
Role of spectral methods	Spectral or SVD techniques are used primarily within the private community recovery subroutine	Private spectral estimation is the central operation used recursively at each hierarchical split
Primary utility criterion	Approximation ratio and additive error with respect to Dasgupta’s cost	Eigenvector estimation error, split or overlap accuracy, and hierarchical recovery
HSBM guarantee	1+o(1) approximation to Dasgupta’s cost and recovery of the underlying blocks under separation conditions	Conditions for exact recovery of the recursive spectral splits and, consequently, the target hierarchy

**Table 2:** List of symbols.

Symbol	Description
G=(V,E)	Undirected graph with vertex set *V* and edge set *E*
*n*	Number of vertices, i.e., |V|
$A\in {\left\{0,1\right\}}^{n\times n}$	Adjacency matrix of the graph, symmetric with zero diagonal
$A_{S}$	Induced adjacency submatrix restricted to node subset S⊆V
$u_{S}^{★}$	Leading eigenvector of $A_{S}$ defining the true binary split
$\Delta_{S}$	Local eigengap, $\Delta_{S}=\lambda_{1}\left(A_{S}\right)-\lambda_{2}\left(A_{S}\right)$
$\rho_{S}$	Contraction ratio, $\rho_{S}=\lambda_{2}\left(A_{S}\right)/\lambda_{1}\left(A_{S}\right)$
*S*	Subset of vertices corresponding to a node in the hierarchy
$S^{+},S^{-}$	Two subsets obtained from binary spectral split of *S*
*k*	Number of leaf (primitive) communities
*L*	Depth of the hierarchical tree, typically $L=\log_{2}k$
$x_{S}^{\left(t\right)}$	Iteration vector at step *t* in the noisy power method
$N_{l}$	Number of power iterations at depth *ℓ*
$\sigma_{l}$	Noise standard deviation at depth *ℓ* for DP mechanism
${\mathrm{err}}_{S}$	Local misclassification rate for split at node *S*

## Data Availability

The original contributions presented in this study are included in the article. Further inquiries can be directed to the corresponding author.

## Appendix A. Additional Details

### Appendix A.1. Privacy Threat Model

We consider a centralized privacy setting where a trusted curator has access to the full graph, including all nodes and edges. The primary source of sensitivity lies in the *relationships* between nodes rather than node attributes, as the presence or absence of an edge may reveal sensitive information about the interactions between individuals or entities. We adopt the standard edge-DP framework, which requires that the output of the mechanism remains stable under the addition or removal of a single edge. The adversary is assumed to possess partial knowledge of the graph structure and aims to infer undisclosed relationships. Our goal is therefore to ensure that the released clustering structure does not reveal sensitive edge information while still enabling accurate recovery of the underlying community organization.

### Appendix A.2. Evaluation Metric

We first evaluate performance at the leaf level of the hierarchy. Let $\sigma^{★}\in {\left\{1,\ldots ,k\right\}}^{n}$ denote the ground-truth leaf labels induced by the leaves of $T^{★}$, and let $\hat{\sigma }\in {\left\{1,\ldots ,k\right\}}^{n}$ denote the estimated labels produced by a clustering algorithm. We define the permutation-invariant error rate as follows:

(A1) $$\mathrm{err}\left(\hat{\sigma },\sigma^{*}\right):=\frac{1}{n}\cdot \min_{\pi \in S_{k}}\mathrm{Ham}\left(\pi \left(\hat{\sigma }\right),\;\sigma^{*}\right),$$

where $S_{k}$ is the set of permutations on {1,…,k} and Ham(·,·) denotes the Hamming distance. Equivalently, we define the confusion matrix $C\in N^{k\times k}$ by

(A2) $$C_{ab}:=\left|\{i\in V:\sigma_{i}^{★}=a,\;{\hat{\sigma }}_{i}=b\}\right|,\;a,b\in \left\{1,\ldots ,k\right\}.$$

Then, the permutation-invariant error admits the representation

(A3) $$\mathrm{err}\left(\hat{\sigma },\sigma^{*}\right)=1-\frac{1}{n}\cdot \max_{\pi \in S_{k}}\sum_{a=1}^{k}C_{a,\pi (a)}.$$

Thus, the leaf-level error is determined by the optimal alignment between the predicted and true clusters through the confusion matrix. Each node i∈V corresponds to a leaf in the hierarchy and is associated with a path from the root to that leaf. We represent this path with a binary vector $h_{i}^{★}\in {\left\{\pm 1\right\}}^{L}$, where ${\left(h_{i}^{★}\right)}_{l}$ indicates whether node *i* belongs to the left or right child at a depth *ℓ*. Similarly, the estimated hierarchy induces a path vector ${\hat{h}}_{i}\in {\left\{\pm 1\right\}}^{L}$. For each depth l=1,…,L, we define the level-wise error as follows:

(A4) $${\mathrm{err}}_{l}:=\frac{1}{n}\min_{s\in \{\pm 1\}}\left|\left\{i\in V:{\hat{h}}_{i,l}\neq s\;h_{i,l}^{★}\right\}\right|.$$

This measures the fraction of nodes whose left or right assignment at a level *ℓ* is incorrect up to the global label symmetry of a binary split. We define the overall hierarchical error as the average of the level-wise errors:

(A5) $${\mathrm{err}}_{\mathrm{hier}}:=\frac{1}{L}\sum_{l=1}^{L}{\mathrm{err}}_{l}.$$

**Proposition A1** 
(Hierarchical error decomposition)**.** *For the hierarchical estimator, the leaf-level permutation-invariant error satisfies*

(A6) $$\mathrm{err}\left(\hat{\sigma },\sigma^{★}\right)\;\leq \;\sum_{l=1}^{L}{\mathrm{err}}_{l}.$$

**Proof.** If node *i* is assigned to an incorrect leaf, then along its estimated root-to-leaf path, there must exist at least one level l∈{1,…,L} at which the binary decision differs from the ground-truth decision up to a global sign flip. Hence, the event where node *i* is misclassified at the leaf level is contained in the union of the events where node *i* is misclassified at level *ℓ*. Applying a union bound and averaging over all nodes yields

$$\mathrm{err}\left(\hat{\sigma },\sigma^{★}\right)\leq \sum_{l=1}^{L}{\mathrm{err}}_{l}.$$

This completes the proof. □

**Table A1:** Example LLM-related sentences used to construct the semantic graph.

**Watermarking:** Watermarking can help detect whether a generated passage came from a language model; a green list-based watermark biases token selection; robust watermark detection should tolerate paraphrasing.
**Privacy:** Differential privacy limits information leakage; private training adds noise; a privacy budget controls the utility-privacy tradeoff.
**RAG:** Retrieval-augmented generation uses external documents; a retriever finds relevant passages; document grounding reduces hallucinations.
**Efficient Inference:** Efficient inference reduces latency; quantization lowers compute cost; speculative decoding accelerates generation.

### Appendix A.3. Brief Background on Spectral Clustering

Spectral methods detect communities in a graph G=(V,E) by exploiting the eigenstructure of its adjacency matrix A. Let $\lambda_{1}\geq \lambda_{2}\geq \ldots \geq \lambda_{n}$ denote the eigenvalues of A, with corresponding eigenvectors $u_{1},u_{2},\ldots ,u_{n}$. In the case of two communities, the informative direction is captured by a leading nontrivial eigenvector of A, which reflects the dominant variation induced by the underlying partition. The entries of this eigenvector provide a one-dimensional embedding of the vertices, and a binary partition can be obtained by thresholding its values (e.g., by their signs), thereby separating the graph into two communities.

**Recursive Spectral Splitting.** In our hierarchical framework, this idea is applied recursively. At each depth *ℓ*, for a subset of vertices S⊆V, we consider the induced adjacency submatrix $A_{S}$. Under the structural spectral condition specified in Section 2.1.3, we estimate the dominant eigenvector $u_{S}^{★}$ of $A_{S}$ via power iteration (or its noisy variant), whose sign pattern defines the target binary split

(A7) $$\begin{array}{cc} S^{+} & =\{i\in S:u_{S}^{★}\left(i\right)\geq 0\}, \end{array}$$

(A8) $$\begin{array}{cc} S^{-} & =\{i\in S:u_{S}^{★}\left(i\right)<0\}. \end{array}$$

This procedure is then applied recursively to the resulting subsets.

#### Auxilary Lemmas

We now specialize the local spectral quantities under the logarithmic-degree HSBM scaling. In this regime, the connection probabilities decay with the node size, and the spectral gap is no longer linear in *m* but instead scales logarithmically. The following lemma characterizes the behavior of the local eigengap at a node *S*.

**Lemma A1** 
(Local eigengap under logarithmic HSBM scaling)**.** *Consider a node S at a depth ℓ with m=|S|. Suppose that the HSBM connection probabilities at consecutive depths satisfy*

$$p_{l}=\alpha_{l}\cdot \frac{\log m}{m},\;p_{l+1}=\alpha_{l+1}\cdot \frac{\log m}{m},$$

*for constants $\alpha_{l+1}>\alpha_{l}>0$. Define the local gap*

$$\alpha_{l+1}-\alpha_{l}.$$

*Then, the population eigengap satisfies*

$$\Delta_{S}^{\mathrm{pop}}≍m\cdot \left(p_{l+1}-p_{l}\right)=\left(\alpha_{l+1}-\alpha_{l}\right)\cdot \log m.$$

*Moreover, there exist universal constants $c,c_{1},c_{2}>0$ such that for sufficiently large m values, with a probability of at least $1-c_{2}m^{-3}$, we have*

$$\Delta_{S}=\lambda_{1}\left(A_{S}\right)-\lambda_{2}\left(A_{S}\right)\geq c\;\cdot \left(\alpha_{l+1}-\alpha_{l}\right)\cdot \log m-c_{1}\cdot \sqrt{\alpha_{l+1}\cdot \log m}.$$

*In particular, if*

$$\left(\alpha_{l+1}-\alpha_{l}\right)\log m\gg \sqrt{\alpha_{l+1}\log m},$$

*then*

$$\Delta_{S}=\Omega \left((\alpha_{l+1}-\alpha_{l})\cdot \log m\right).$$

We next combine the above eigengap characterization with the local noisy power iteration bound to obtain an explicit error guarantee in terms of the HSBM parameters. This result highlights how the estimation error depends on the local separation between consecutive levels of the hierarchy.

**Corollary A1** 
(Local noisy power error under logarithmic HSBM scaling)**.** *Consider a node S at a depth ℓ with m=|S|. Suppose that*

$$p_{l}=\alpha_{l}\cdot \frac{\log m}{m},\;p_{l+1}=\alpha_{l+1}\cdot \frac{\log m}{m},\;\alpha_{l+1}>\alpha_{l}>0,$$

*and assume that the local eigengap satisfies*

$$\Delta_{S}=\Omega \left((\alpha_{l+1}-\alpha_{l})\cdot \log m\right).$$

*Run the local noisy power method with*

$$\sigma_{l}=\frac{\sqrt{4N_{l}\log\left(1/\delta_{l}\right)}}{\varepsilon_{l}},\;N_{l}=O\left(\log m\right).$$

*Then, with a probability of at least $1-\eta -c_{2}m^{-3}$, we have*

$$\min_{s\in \{\pm 1\}}{∥x_{S}^{\left(N_{l}\right)}-su_{S}^{★}∥}_{2}≲\frac{\sqrt{m\log m\;\log(1/\delta_{l})}}{\varepsilon_{l}\left(\alpha_{l+1}-\alpha_{l}\right)\log m}.$$

*Equivalently, we have*

$$\min_{s\in \{\pm 1\}}{∥x_{S}^{\left(N_{l}\right)}-su_{S}^{★}∥}_{2}≲\frac{\sqrt{m\;\log(1/\delta_{l})}}{\varepsilon_{l}\left(\alpha_{l+1}-\alpha_{l}\right)\sqrt{\log m}}.$$
